# Case Report: Virtual surgery and 3D printing in a medication-related osteonecrosis of the jaws (MRONJ) pathological mandibular fracture

**DOI:** 10.3389/froh.2025.1520195

**Published:** 2025-03-28

**Authors:** Giorgio Lo Giudice, Alessandro Calvo, Emanuele Magaudda, Francesco Saverio De Ponte, Enrico Nastro Siniscalchi

**Affiliations:** ^1^ Department of Biomedical and Dental Sciences and Morphofunctional Imaging, Maxillo-Facial Surgery Unit, University Hospital “G. Martino”, University of Messina, Messina, Italy; ^2^ Department of Medicine and Surgery, University of Enna “Kore”, Enna, Italy

**Keywords:** CAD/CAM, 3D printing, facial fracture, MRONJ, medication-related osteonecrosis of the jaw, virtual surgery

## Abstract

**Introduction:**

The use of anatomical models, guides, and surgical templates allows for increased precision of interventions and reduced operative times. Thanks to computer-aided design (CAD) and computer-aided manufacturing (CAM) technologies and rapid prototyping through 3D printing, it is possible to obtain accurate models, which are useful to defining surgical planning in the maxillofacial district.

**Methods:**

We present the case of a patient with a pathological fracture of the mandibular body affected by medication-related osteonecrosis of the jaws (MRONJ) in stage III. Through the manipulation of virtual models obtained from thin-layer Computed Tomography (CT), a virtual surgical intervention of sequestrectomy and debridement of necrotic bone tissue, reduction and containment of the fracture was performed. The resulting mandibular model was used as a template for the preoperative modeling of the titanium reconstruction plate used for fracture containment.

**Results:**

The intraoperative result and follow-up demonstrated good accuracy of the model with respect to post-operative mandibular dynamics, condylar-fossa position and a reduced surgical time.

**Discussion:**

Virtual surgery and 3D-printed prototyping represent a feasible technique in MRONJ patients, allowing increased precision of interventions, reduced risks associated with the operation, and improved operative and recovery times for the patient.

## 1 Introduction

The recommended guidelines on medication-related osteonecrosis of the jaws (MRONJ) treatment have been evolving in the last decades (1–5). MRONJ treatments can be divided into conservative and surgical approaches. The first AAOMS recommendations emphasized conservative approaches, focusing on symptom resolution and avoiding invasive interventions in compromised patients. Conservative treatment involves managing the bacterial environment with broad-spectrum antibiotics, such as amoxicillin/clavulanic acid and metronidazole, alongside oral antiseptics like chlorhexidine. Non-invasive treatments like ozone, laser, and hyperbaric therapy may complement antibiotics, though their benefits are not definitively proven. Overall, while conservative treatment aims to control infection and prepare for possible surgery, more invasive surgical interventions are gaining traction for better outcomes.

Surgical treatment for MRONJ has evolved from being limited to advanced stages to being considered even in selected Stage I cases. The 2022 AAOMS update aligns with other international guidelines, emphasizing that MRONJ is a focal bone pathology. Adequate removal of affected bone can lead to disease resolution and improved quality of life, with a 10% increase in survival rate two years post-diagnosis. Surgical interventions are categorized by the extent of necrotic bone resection and removal of possible infection reservoirs, aiming for healthy and viable margins and soft tissue healing (6, 7).

In the era of the digital workflow revolution and personalized medicine, Computer-Aided Design/Computer-Aided Manufacturing (CAD/CAM) approaches to maxillofacial traumatology and oncology have become the norm (8). While is yet to be found a precise method to minimally approach MRONJ cases, ensuring at the same time a resolutive surgery and balancing the innate fragility of these patients, the aim of this case is to describe a case of Stage 3 MRONJ that was treated using a full digital workflow, by the means of virtual surgery and a pre-bent reconstructive plate on a 3D printed model.

## 2 Case description

The study was conducted in adherence with the Declaration of Helsinki Principles and was approved by the ethics committee (prot. 95-22, 05/09/2022, AOU Policlinico “G. Martino”, Messina) and following the CARE guidelines. Informed consent was obtained from all subjects involved in the study.

**Figure 1:**
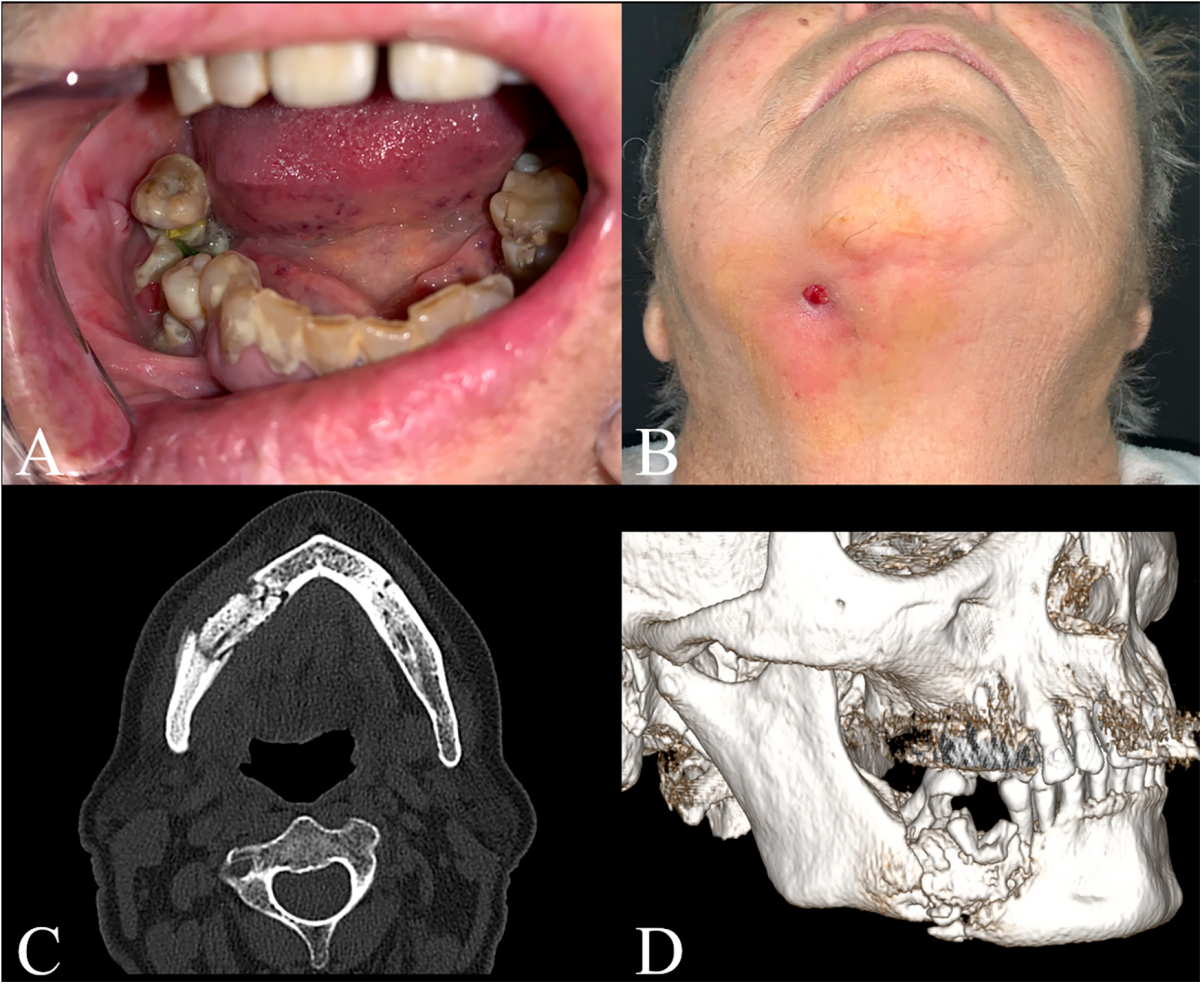
Patients’ clinical examination and preoperative CT scan. **(A)** Intraoral view; **(B)** Submentovertex view; **(C)** Axial view; **(D)** 3D reconstruction.

A 76-year-old patient presented at the emergency room of the Policlinico G. Martino in Messina complaining of severe pain and swelling in the right hemimandibular area. At the physical examination, the patient presented a partially edentulous lower quadrants and evidence of endo-oral bone exposure in the fourth quadrant and right hemimandibular oro-cutaneous fistula (Figures 1A,B). The patient had positive pharmacological history for Doryx (Alendronic Acid, SF Group S.r.l.) which she had been taking for 10 years for osteoporosis treatment, uncontrolled insulin-dependent Type 2 diabetes and dementia. A thin-layer Computed Tomography (CT) scan was performed which showed a large sequestration of the right hemimandible with a fracture of the homolateral mandibular body, confirming the diagnosis of Stage III MRONJ (Figures 1C,D). The patient was then transferred to the Maxillofacial Surgery department for treatment. The patient began IV antibiotic therapy consisting of amoxicillin/clavulanic acid 1.75 g/250 mg and metronidazole 1.5 g daily, and oral rinses with 0.2% Chlorhexidine solution. This therapy started at time of admission prior surgery, was continued during the hospital stay (7 days) and for 15 days at home.

A 3D virtual model of the mandible was generated from DICOM images obtained from CT scans, using the open-source software 3D-Slicer ® (9).

Virtual surgery was then conducted on this model. Bone segmentation was performed via thresholding within the Segment Editor module, isolating the mandible. Guided by CT slices, a virtual sequestrectomy and debridement were manually executed using the Cut function, focusing on the identification of sequestrum, trabecular bone structural changes, osteosclerosis, erosions, and microlacunae. The resulting mandible stump models were then created.

**Figure 2:**
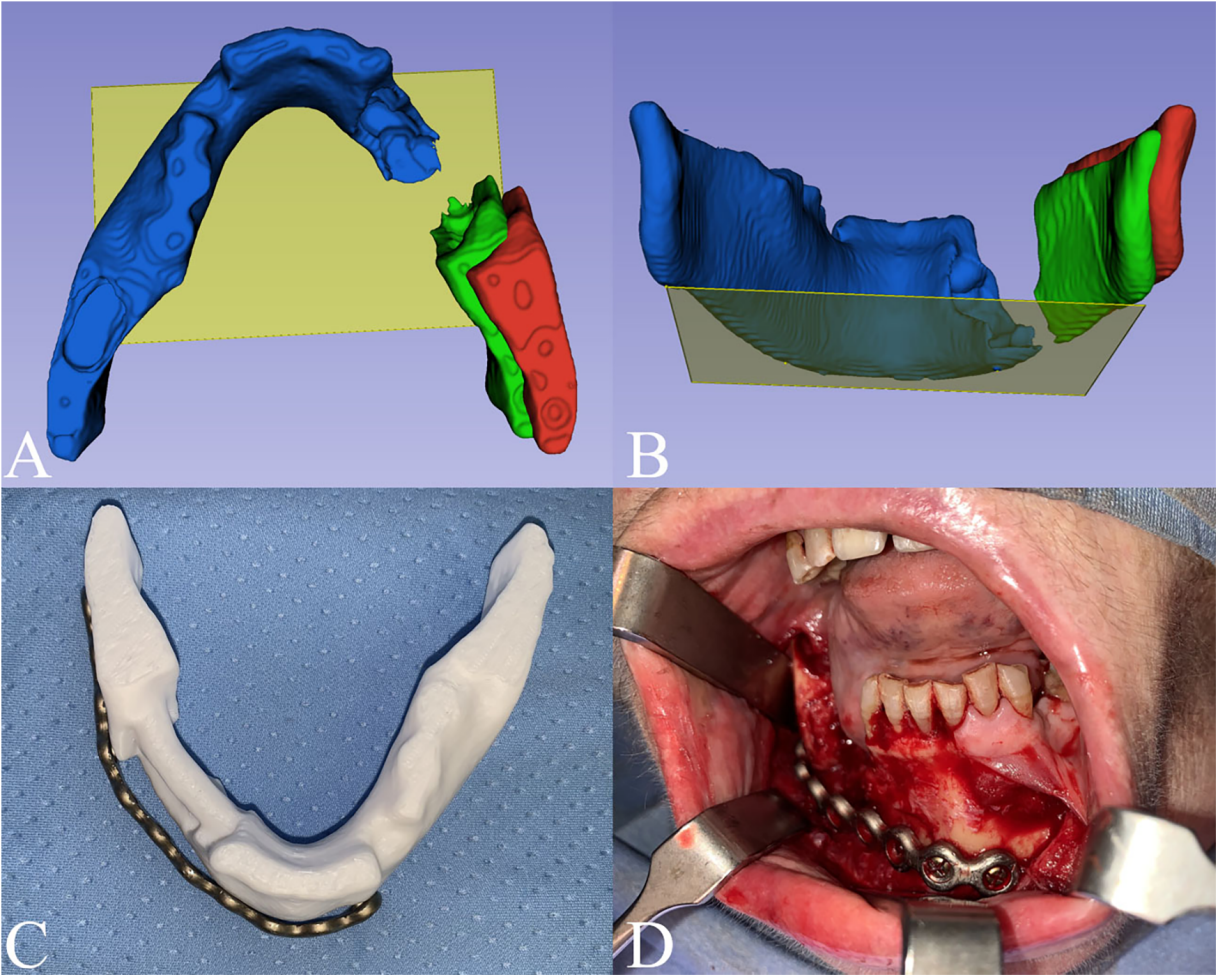
Digital planning. The fracture reduction was performed using as a reference a plane (yellow plane) tangent to 3 points on the lower margin of the ramus and symphysis on the healthy left mandible stump (blue). The right mandible stump (red) was then rotated and translated into its final position (green). **(A)** Axial view; **(B)** Postero-anterior view. **(C)** Titanium plate preplating on the planned model; **(D)** intraoperative setting of the prebent plate.

To reconstruct the mandibular arch, transformations were applied to reduce the fracture. A reference plane, created using the Markups module, was defined by three points tangent to the lower margin of the ramus and symphysis of the healthy left mandible stump, with respect to the condylar-fossa position. The right mandible stump was subsequently rotated and translated into its correct position using the Transform module (Figures 2A,B). Finally, to ensure stability for 3D printing, the right mandibular gap was bridged.

A physical model was produced by 3D printing with PLA + filament, using Snapmaker Original 3D printer (Snapmaker ®, Shenzhen, China). The printed model was then used to guide the preoperative preplating of the titanium reconstruction plate used for fracture containment. The pre-bent titanium plate was then sterilized as per Hospital protocols (Figures 2C,D).

The surgical procedure foresaw 3 steps:

(1) An intraoral approach to the mandible was performed. The 2.5 mm titanium reconstruction plate was firmly positioned in place thanks to the guidance of the preoperative planning and chin-side curve of the plate. Screw holes were pre-dilled to guide the subsequent fixation.
(2) Sequestrectomy and debridement were performed through piezosurgery. It consisted of necrotic bone removal achieving macroscopically healthy margins ensured by bleeding of the surrounding bone, followed by the closure of the oro-cutaneous fistula in multiple layers using Vicryl 3.0.
(3) Mandible fixation was performed using the pre-bent titanium plate and screws. Mandibular dynamics were evaluated and were deemed as preserved. Tensionless suture of the mucosal layer was performed using Vicryl 3.0.

**Figure 3:**
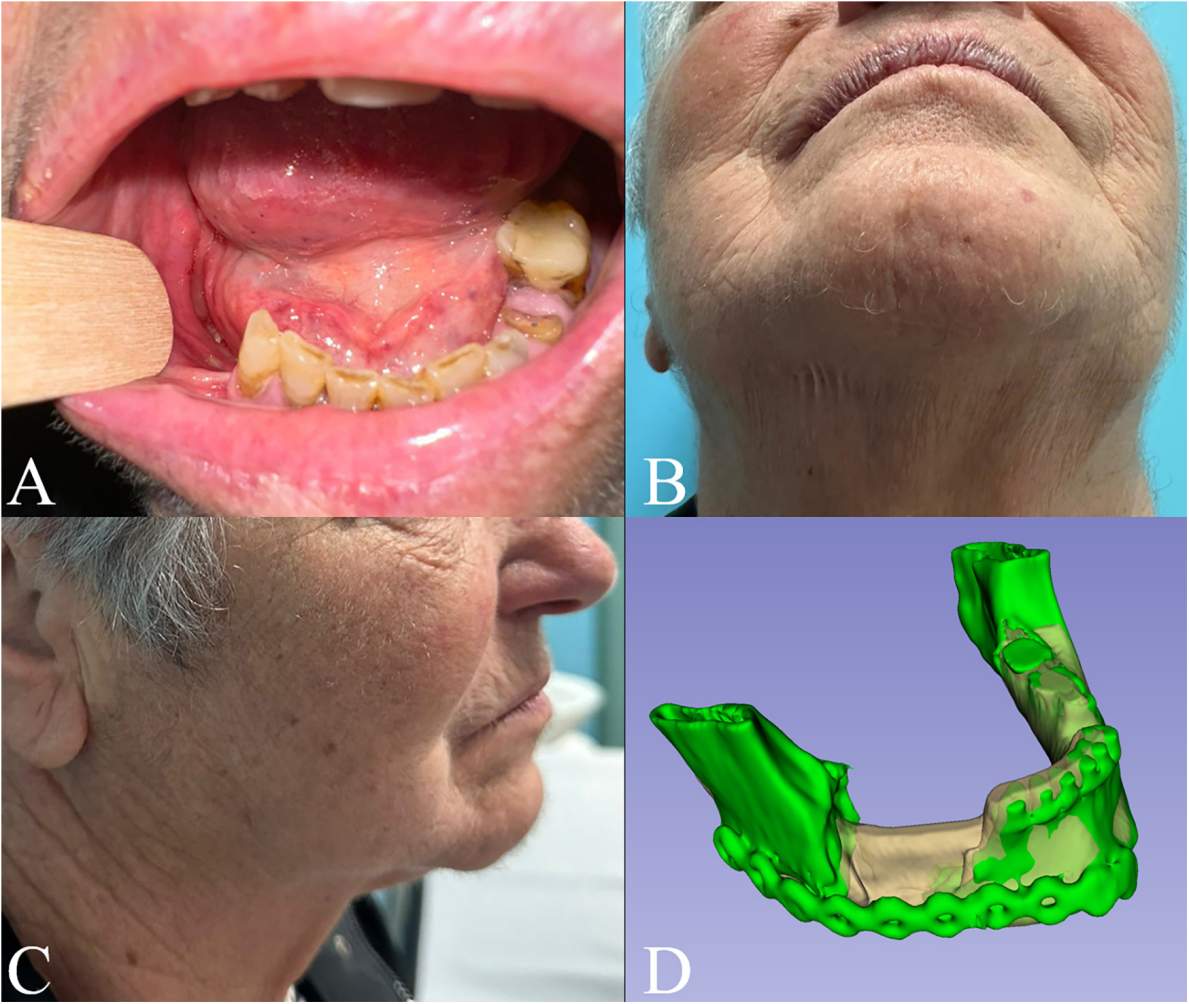
6 months follow-up in **(A)** intraoral, **(B)** submentovertex and **(C)** right lateral view; **(D)** superimposition of the preoperative model (white) and the postoperative one (green).

Postoperative CT scan model was created and superimposed to the virtual model to assess planning reliability and the consistency of the intraoperative excision borders with the ones virtually planned (Figure 3). The patient, fed through nasogastric tube during the recovery, had an uneventful postoperative case and was discharged home after 7 days. Careful counseling was carried out to educate the patient and caregivers to the best home oral hygiene (using antimicrobial oral rinses with 0.2% chlorhexidine solutions and tooth brushing) and strict glycemic control.

**Figure 4:**
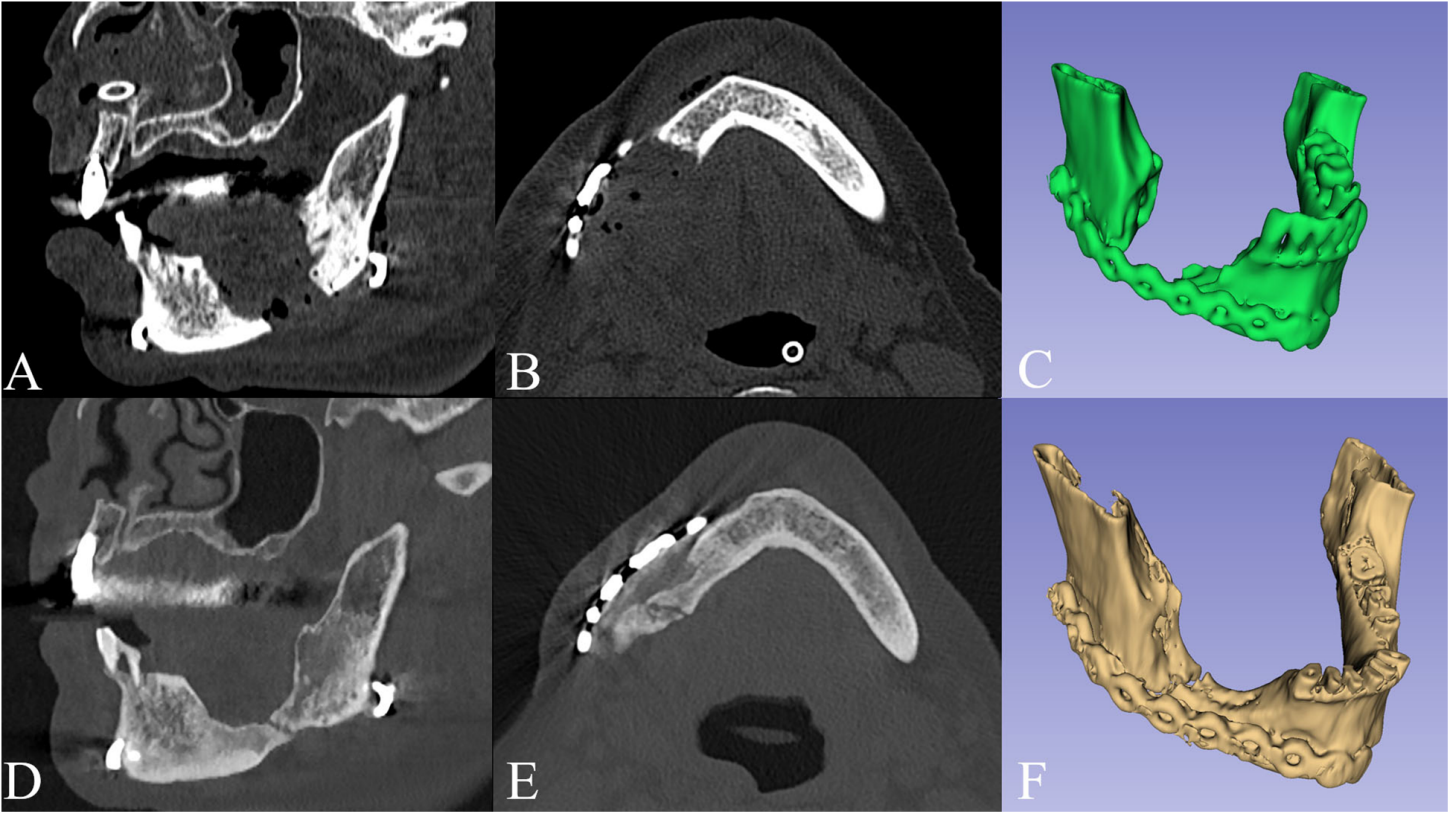
Sagittal view **(A)**, axial view **(B)** and 3D model **(C)** of the postoperative CT scan, compared to the 2 years follow-up **(D**–**F)**, showing bone regeneration at the lower mandibular margin within the surgical gap.

Intra- and extra-oral stability of the outcomes was observed at six-month follow-up (Figure 3). Notably, two-year Dental Scan CT images confirmed bone regeneration at the lower mandibular margin within the surgical gap (Figure 4).

## 3 Discussion

Both the American and Italian boards advocate for a patient-specific evaluation of the cost–benefit ratio, regardless of disease stage. They suggest prioritizing the achievement of healthy and viable bony margins over the traditional principle of avoiding invasiveness to protect quality of life. Meticulous planning is essential and can greatly influence the procedure's success (10). Nonetheless, high stage MRONJ cases are often complex to treat adequately. The patients tend to be advanced in age, have poor general conditions, several comorbidities, and are unable to undergo intensive surgeries for an extended period of time. Precision medicine holds the key to overcoming these challenges.

A crucial topic is achieving safe surgical margins, as there remains no consensus on the gold standard for assessing these margins radiographically or intraoperatively (11). The choice of imaging modality is influenced not only by the surgeon's preference but also by the available options. For extended resections and reconstruction planning, a three-dimensional imaging modality is highly desirable and, in severe cases, essential. Panoramic radiographs routinely used for dental assessments are often insufficient, requiring additional diagnostics. Cone Beam Computed Tomography (CBCT) has shown to be superior to panoramic radiographs in detecting fragmentation, sequestra, pathological fractures and marrow space narrowing, granting at the same time low radiation dosage (12). CT scans offer detailed assessments detecting structural bone alterations, cortical bone erosion, and trabecular bone resorption, crucial for accurate diagnosis, and can show recurrent disease signs within six months post-surgery (13). Magnetic Resonance Imaging (MRI) is valuable for early detection, distinguishing between osteonecrotic and osteomyelitic patterns, showing markedly decreased T1 signals and increased T2 signals, with contrast uptake in affected areas (14). However, Chiandussi et Al. showed that 99Tcm-MDP 3-phase bone scan can be more effective in defining disease extent than both MRI and CT (15). While MRI/CT fusion is often recommended, its routine application remains uncertain in terms of cost-benefit ratio and poses logistical challenges (16).

Applications of functional imaging techniques are being more frequently studied to evaluate MRONJ. SPECT/CT imaging provides detailed 3D images by monitoring radionuclide distribution. MRONJ should not show uptake in the necrotic zone; however, infection-related uptake may occur (11). Scintigraphy has potential prognostic value and can predict MRONJ development. PET/CT can detect metabolic changes not visible in plain radiography, but it cannot identify aseptic necrosis (17, 18). Some studies suggest using doxycycline and tetracycline fluorescence to assess bone vitality, but these methods are still experimental (19, 20).

Overall, a combination of these imaging modalities provides a comprehensive diagnostic and management approach for MRONJ patients. The expertise of the surgeon is crucial, as methods like intraoperative observation of bleeding margins lack definitive reliability given that macroscopically visible necrotic bone often correlates with varying degrees of surrounding osteomyelitis and soft tissue involvement (21, 22).

While using different imaging modalities can help in diagnosis, the choice may impact CAD/CAM workflow. CBCT and CT scans are in general easily segmented, manually or automatically, to create models and apply changes to them. On the other hand, there is a notable lack of automated software for MRI bone segmentation, unlike the available tools for CT images. Bone segmentation on MRI is predominantly manual or requires extensive manual editing, though some semi-automated methods like thresholding, region growing, or ray casting are applicable. The challenge in developing fully automated segmentation arises from nearby structures sharing the same intensity as bone, leading to inaccuracies. Special cases like synthetic CT (sCT) images can use HounsfieldUnits-based segmentation benefiting from CT-dedicated software. MRI segmentation is generally more time-intensive than CT, sometimes taking more than twice the time. This will possibly be mitigated by automated deep learning methods currently in development (16). Therefore, to be a CT surrogate for bone visualization, MRI should provide images on which bone can be segmented within time and with a level of accuracy similar to or better than what can be achieved on CT.

Current approaches recommend evaluating the cost–benefit ratio of surgery on a patient-specific basis, regardless of disease stage. Particularly in early stages, conservative resection can achieve healthy bone margins, preserve anatomy and improve success rates. In advanced stages, significant resections may be necessary, potentially leading to debilitating outcomes and requiring reconstructive procedures. Limited literature exists on flap reconstruction with vascularized bone (23, 24). Vascularized flaps allow for prosthetic dental rehabilitation but may be overtreatment given the typically short life expectancy of MRONJ patients and high comorbidity rate, favoring less invasive approaches. Less invasive options, like reconstruction plates with or without loco-regional flaps, are also considered and usually preferred.

Studies on preplating or pre-adjusted plates indicate their superiority over conventional 3D plating, and they can be effectively incorporated also for comminuted fractures (25–28). These benefits include fewer bends, shorter fixation times, and reduced pain during adaptation. Advantages observed include restored facial symmetry and function, corrected orbital occlusion, resolution of enophthalmos and diplopia, and cosmetically symmetrical lower face reconstruction. Although three-dimensional printed models reduce surgery time, they require longer preparation and higher production costs. However, in-house production options mitigate these time and cost factors (29).

Regarding displaced mandibular fractures, one of the challenges is to plan the fixation with respect to the original condylar-fossa position, and to restore a correct mandibular dynamic. Although using occlusal guidance or maneuvers on the distal stump can be employed to do so, drawing from orthognathic surgery experience, these options are not feasible for edentulous or partially edentulous patients (30). In our case, the position of the distal stump of the fracture was checked during the virtual planning, then evaluated intraoperatively after fracture fixation. Planning accuracy was also evaluated by the superimposition the preoperative model with the model coming from the postoperative CT scan, also to exclude that muscular forces could disrupt the static or dynamic position, showing good results (Figure 3).

The use of anatomical models, guides, and surgical templates allows for increased precision of interventions and reduced operative times. In particular, virtual anatomical models obtained from thin-layer CT and processed using 3D modeling software allow for accurate visualization of the morphology of the maxillofacial district and the execution of virtual surgical interventions in a precise and detailed manner (31). 3D prototyping through 3D printing allows for the rapid and economical production of physical anatomical models, which can be used as templates for the preoperative modeling of devices such as reconstruction plates, surgical guides, and prostheses (8, 32). These models can faithfully reproduce anatomy, allowing surgeons to plan the intervention in a precise and personalized manner (33).

This full digital protocol presents a cost-effective alternative to traditional methodologies. The fabrication of models is economical, with raw material costs for the model produced in this case approximating €0.30–€0.40, and utilizes free, open-source software. Model creation was relatively rapid, requiring around 3 h to complete. The titanium plates employed are readily available within a standard Maxillofacial unit, further containing expenses. Pre-bent plates allow for some intraoperative flexibility; however, modifications during surgery are constrained by the pre-planned virtual procedure.

In contrast, custom-made titanium plates or prostheses frequently involve significantly higher production costs due to specialized manufacturing processes and materials. Furthermore, the traditional workflow for custom devices often entails longer fabrication times, requiring additional appointments and potentially delaying treatment. Moreover, unforeseen anatomical variations or surgical complications that may necessitate deviations from the plan are difficult to accommodate with pre-fabricated prostheses.

Despite the numerous advantages offered, the protocol presented is not without limitations. A significant challenge lies in the manual segmentation and fracture realignment process. This process can be particularly demanding and time-consuming, especially when dealing with complex anatomical structures. This segmentation bottleneck can increase the overall time required for preoperative planning, potentially offsetting some of the time-saving benefits of the digital workflow. Regarding the “virtual debridement” phase, despite being performed under CT imaging guidance, safe surgical margins achievement remains a discussed topic.

Furthermore, the accuracy and reliability of the final surgical outcome are highly dependent on the precision of each step in the digital process. Errors introduced during segmentation, virtual planning, or 3D printing can propagate and ultimately affect the fit of the pre-bent plates. The technical skills and expertise required to effectively utilize the software and hardware components of the digital protocol also represent a limitation. A learning curve is associated with mastering CAD/CAM software and 3D printing technology, and inadequate training can lead to suboptimal results.

Although this study focused on a single case, the promising results suggest how virtual surgery and 3D prototyping can play a significant role in the surgical planning of patients affected by MRONJ.

## 4 Conclusions

Virtual surgery and 3D-printed prototyping represent a viable and practical workflow in MRONJ patients, allowing increased precision of interventions, reduced risks associated with the operation, and improved operative and recovery times for the patient. This digital workflow is also easily applicable to facial fractures of different etiology respecting the symmetry planes and condylar-fossa relationship in mandibular fractures.

## Data Availability

The raw data supporting the conclusions of this article will be made available by the authors, without undue reservation.
